# Neuroinflammation as a Key Driver of Secondary Neurodegeneration Following Stroke?

**DOI:** 10.3390/ijms222313101

**Published:** 2021-12-03

**Authors:** Shannon M. Stuckey, Lin Kooi Ong, Lyndsey E. Collins-Praino, Renée J. Turner

**Affiliations:** 1 Discipline of Anatomy and Pathology, School of Biomedicine, Faculty of Health and Medical Sciences, The University of Adelaide, Adelaide 5005, Australia; shannon.stuckey@adelaide.edu.au (S.M.S.); lyndsey.collins-praino@adelaide.edu.au (L.E.C.-P.); 2 School of Pharmacy, Monash University Malaysia, Subang Jaya 47500, Malaysia; ong.linkooi@monash.edu; 3 School of Biomedical Sciences and Pharmacy and the Priority Research Centre for Stroke and Brain Injury, The University of Newcastle, Callaghan 2308, Australia

**Keywords:** stroke, cerebral ischaemia, dementia, inflammation, microglia, astrocytes, anti-inflammatories, neuroprotection, therapeutic

## Abstract

Ischaemic stroke involves the rapid onset of focal neurological dysfunction, most commonly due to an arterial blockage in a specific region of the brain. Stroke is a leading cause of death and common cause of disability, with over 17 million people worldwide suffering from a stroke each year. It is now well-documented that neuroinflammation and immune mediators play a key role in acute and long-term neuronal tissue damage and healing, not only in the infarct core but also in distal regions. Importantly, in these distal regions, termed sites of secondary neurodegeneration (SND), spikes in neuroinflammation may be seen sometime after the initial stroke onset, but prior to the presence of the neuronal tissue damage within these regions. However, it is key to acknowledge that, despite the mounting information describing neuroinflammation following ischaemic stroke, the exact mechanisms whereby inflammatory cells and their mediators drive stroke-induced neuroinflammation are still not fully understood. As a result, current anti-inflammatory treatments have failed to show efficacy in clinical trials. In this review we discuss the complexities of post-stroke neuroinflammation, specifically how it affects neuronal tissue and post-stroke outcome acutely, chronically, and in sites of SND. We then discuss current and previously assessed anti-inflammatory therapies, with a particular focus on how failed anti-inflammatories may be repurposed to target SND-associated neuroinflammation.

## 1. Introduction

Stroke involves the rapid onset of focal neurological disturbances such as paralysis, impaired speech, or loss of vision due to a disruption in cerebral blood flow [1,2]. Ischaemic strokes, which are due to an occlusion in the cerebral blood vessels, are more common than haemorrhagic strokes, accounting for approximately 87% of all stroke cases [3]. One in four people will suffer a stroke in their lifetime [4], which equates to over 17 million strokes worldwide each year, with over two thirds of these cases resulting in death or severe disability [5]. Furthermore, the absolute number of people dying from stroke and the total number of disability-adjusted life-years lost due to stroke has increased since 1990, with stroke now the second leading cause of death and leading cause of acquired disability worldwide [6]. Even in individuals who survive the initial stroke event, there is an increased risk for the development of a number of persistent complications, including motor disability [7,8], speech deficits [9,10], cognitive impairment [11,12,13,14,15,16] and fatigue [17,18,19,20], amongst many others. In particular, cognitive impairment is observed in a large proportion of patients post-stroke, even in those with successful clinical recovery (no significant disability) [12]. Such post-stroke cognitive impairment has been implicated in the worsening of functional outcomes, including the development of long-term depression and post-stroke dementia (PSD) [21,22,23]. In fact, stroke is a significant risk factor for dementia, with approximately 30% of stroke patients going on to develop it [24].

While the neural mechanisms that link stroke to the later development of dementia remain unclear, accumulating evidence has shown that neuronal degeneration can occur in distal areas of the brain several weeks to months post-stroke [25,26,27]. This phenomenon, termed secondary neurodegeneration (SND), involves the progressive death of neurons in distal regions of the brain that are anatomically connected to the site of infarction, but which were not initially affected by the reduction in cerebral blood flow brought on by the initial stroke [28]. SND has consistently been observed in both clinical neuroimaging studies [25,29,30] and in experimental studies at the cellular level [31,32,33]. Various sites appear to be affected by SND, dependent on their connectivity with the infract site, as well as other cortical structures undergoing degeneration. However, from the literature to date, the thalamus, hippocampus and basal ganglia appear to be the most readily affected [25,34,35]. Particularly, a magnetic resonance imaging (MRI) study demonstrated evidence of SND in the thalamus of patients three months after an ischaemic insult [29], whereas another study noted thalamic degeneration on MRI within the first few weeks post-stroke, with one patient demonstrating degeneration in the ventral nuclei as early as eight days following an ischaemic stroke [25]. In line with this, a recent neuroimaging study reported atrophy of the caudate nucleus, putamen, pallidum and thalamus following middle cerebral artery (MCA) stroke in patients more than three weeks post-stroke [30]. Interestingly, the authors demonstrated that localization of the infarct significantly influenced the secondary changes seen in the basal ganglia and thalamus, as atrophy of these structures was not seen following anterior cerebral artery or posterior cerebral artery stroke [30]. Interestingly, a similar study 15 months post-stroke found that degeneration of the hippocampus was strongly associated with impairment of learning, memory and cognitive function in post-stroke dementia patients [36]. Moreover, recent research demonstrated that the rate of atrophy in regions such as the thalamus and hippocampus following clinical stroke was greater than that seen in Alzheimer’s (AD) patients, suggesting accelerated degeneration following stroke and thus further highlighting a link between SND and onset of dementia [37].

Various pathological changes have been implicated in sites of secondary neuronal damage [38,39,40,41]. Concerningly, it appears that sites of SND also share pathophysiological features with other neurodegenerative conditions, including disturbances in amyloid-beta (Aβ) and hyperphosphorylated tau [42,43,44,45,46,47,48]. In particular, recent evidence has suggested that glial activation and pro-inflammatory cytokine release may stimulate the deposition of Aβ and hyperphosphorylated tau in both stroke and dementia pathology [49,50,51]. This observation is of particular interest, given that neuroinflammation has been found to be a significant driver in the post-stroke secondary neurodegenerative process, suggesting that a persistent neuroinflammatory response, which does not resolve after the initial insult, may play a key role in worsened long-term outcomes, specifically, the latter onset of dementia [13,52,53,54].

As such, in this paper we sought to present a review of the current understanding of post-stroke neuroinflammation, with a particular focus on how it pertains to SND and PSD. In this paper, we summarise the pathological changes associated with the cellular and molecular inflammatory changes and highlight the findings of neuroinflammatory biomarkers in post-stroke disorders. We also discuss studies that have investigated outcomes following modulation of the post-stroke inflammatory response, and how stroke clinical trials may be adjusted to attain more favourable long-term outcomes.

## 2. Acute Ischaemic Injury Cascade

When the arterial blood supply to the brain is blocked following an ischaemic stroke, it results in severe focal hypoperfusion, commonly referred to as the region of stroke primary injury [55]. This deprivation of vital oxygen and nutrients that the brain needs to survive, results in a region of neuronal cell death and unsalvageable tissue called the infarct core [56]. Surrounding this zone is the penumbra, an area of minimally perfused cells with a decreased cerebral blood flow that falls below the functional threshold, but just above the level of cellular death [57]. Penumbral tissue in this zone can remain viable for several hours [58], such that timely reperfusion to restore blood flow can rescue these cells from death and prevent expansion of the infarct core [59]. Indeed, the discovery of potentially salvageable penumbral tissue, which can be identified with computerised tomography (CT) perfusion or MRI, is the basis for current reperfusion therapies in tissue plasminogen activators (tPA) and mechanical thrombectomy [60]. The initial ischaemic event sets in motion secondary injury mechanisms which evolve over time and includes factors such as oxidative stress, excitotoxity, calcium dysregulation, cortical spreading depression, blood-brain barrier (BBB) breakdown, cerebral oedema and neuroinflammation, amongst many others. All these secondary injury factors have been well documented to exacerbate the initial tissue damage and lead to infarct expansion (for reviews, see Lo et al., 2003 [61]; Moskowitz et al., 2010 [2]; Xing et al., 2012 [62]).

Immediately following stroke onset, levels of adenosine triphosphate (ATP) drop, due to an imbalance between consumption and synthesis, as the levels of oxygen substrate needed for aerobic ATP production decrease [56,62,63]. Insufficient ATP rapidly leads to a loss of neuronal ionic homeostasis, thereby allowing the movement of sodium into cells, followed by water, which causes cellular swelling (oedema) and shrinking of the extracellular space [2,63]. The loss of ionic homeostasis also leads to the accumulation of glutamate in the extracellular space [56]. This build-up of glutamate then overstimulates α-amino-3-hydroxy-5-methyl-4-isoxazole propionic acid (AMPA) and ionotropic N-Methyl-D-aspartate (NMDA) glutamate receptors, causing an excessive influx of calcium, which, in turn, activates deleterious phospholipases and proteases that degrade key cellular components such as membranes and proteins [62]. Furthermore, elevated levels of both intracellular calcium and sodium drive mitochondrial dysfunction, further exacerbating the bioenergetic crisis, and also the production of reactive oxygen species (ROS) [56,61]. The subsequent imbalance between ROS and endogenous antioxidant defences in the brain results in oxidative stress and marked cellular injury and/or death via degradation of proteins, nucleic acid and carbohydrates [56,62]. Additionally, ROS can promote neuroinflammation by increasing the permeability of the BBB, allowing subsequent entry of peripheral immune cells [2,56]. Conversely, glutamate produced during cerebral hypoxia stimulates lactate dehydrogenase to convert pyruvate to lactate in astrocytes [64]. According to the astrocyte-neuron lactate shuttle, first proposed by Pellerin and Magistretti, lactate that is released from astrocytes is then supplied to nerve cells where it is converted back to pyruvate and directly used to generate energy [65,66]. As such, various studies have noted that lactate released from astrocytes serves as a signal that regulates survival, function, and memory formation through the recovery of damaged neurons [67,68].

Whilst a more in-depth discussion of this multi-faceted cascade is outside the scope of the current review (for reviews see, Brouns and Deyn, 2009 [56] Dirnagl, Iadecola & Moskowitz, 1999 [69]), in recent years, increasing evidence has highlighted the critical role that neuroinflammation plays in driving the secondary injury process.

## 3. Acute Neuroinflammation in Stroke

A robust neuroinflammatory reaction follows cerebral ischaemia, and involves the activation of resident inflammatory cells, the subsequent release of inflammatory mediators, and both the migration and recruitment of leukocytes across the BBB [70,71]. Post-stroke, the neuroinflammatory response is initiated and perpetuated in the brain by numerous aspects of the ischaemic injury cascade, including necrotic cells, debris and ROS [56,72]. One such aspect is the impairment of endothelial nitric oxide synthase which leads to further increases in ROS due to impaired nitric oxide (NO) generation [73]. Increased generation of ROS triggers pro-inflammatory gene activation [73]. Once activated, inflammatory cells, such as microglia and astrocytes, secrete cytokines and chemokines, as well as matrix metalloproteinases (MMPs), NO and more ROS [56,70,72]. Cytokines and chemokines upregulate adhesion molecules, which facilitate the adhesion of leukocytes to the endothelium and their subsequent migration into the brain parenchyma [72,74]. Together, these processes exacerbate cell damage and cause further disruption to the BBB, such as the dysfunction of the endothelium due to apoptosis of endothelial cells and detachment of pericytes [72,73,75,76]. Specifically, within 2 h following cerebral ischaemia, pericytes have been shown to detach from brain microvessels, thereby weakening the interactions between endothelial cells and pericytes [76], which can further contribute to secondary ischaemic damage by permitting blood to enter the brain [70,72]. This sets in motion a cascade of consequences, such as cerebral oedema, microvascular stasis and further infiltration of peripheral inflammatory cells, all of which amplify inflammatory signals and enhance brain tissue damage [70,72]. Indeed, blocking various aspects of the inflammatory cascade has been shown to reduce infarct volume in experimental stroke models [77,78,79]; however, this has yet to be replicated in human stroke patients [71,80,81]. Interestingly, the acute inflammatory response post-stroke can also be beneficial to the ischaemic environment, as it facilitates tissue repair by secreting neurotrophic factors and by clearing debris [82,83,84]. Specifically, astrocytes support neuronal regeneration through the delivery of trophic factors and lipids. They also form the glial scar that walls off the infarction and restricts the spread of inflammation [85]. Moreover, suppression of inflammatory cytokines, such as tumor necrosis factor-alpha (TNF-α) and interleukin (IL)-6, have been shown to worsen post-stroke outcomes in experimental models, likely due to the fact that both cytokines have defined beneficial roles, specifically pertaining to neuronal plasticity [86,87]. The precise nature of these dual properties of inflammation are still not fully understood and, as such, is likely the reason that clinical trials in the acute phase have failed in the past [80,88].

### 3.1. Microglia

Microglia are resident immune cells of the central nervous system (CNS), and they play a critical role in immunological surveillance and have phagocytic functions [89]. After an ischaemic insult to the brain, microglia are rapidly activated (within hours) [72,75,90] and accumulate at the lesion site and within the surrounding penumbra [75,90]. Specifically, 1 h after MCA occlusion (MCAo) in a rat, microglia were not yet observed within the infarct core, but instead were seen within the surrounding penumbral tissue [91]. Microglial cells were first evident in the ischaemic core at 2–4 h post-injury, continuing to increase in number between 70 and 166 h after reperfusion. Notably, during this time, highly ramified microglia were present in the surrounding penumbra, where they continued to increase in both number and intensity [91], suggesting that microglia migrate from the penumbra to the ischaemic core post-stroke. Such microglial findings have also been reported clinically [92,93,94,95,96]. In post-mortem brain tissue, co-expression of pro-inflammatory cytokines with microglia/macrophages was found in the white matter and cerebral cortex as early as 33 h post-stroke and as late as 40 days [97]. Moreover, using positron emission tomography (PET) and T1-weightedMRI, Gerhard et al. measured increased [11C]-PK11195 binding, a ligand selective for translocator protein (TSPO) which is particularly abundant on activated microglia within the infarcted hemisphere of stroke patients as early as day 3 and even out to day 53 post-stroke [92,93]. Similarly, Price et al. investigated ischaemic stroke patients at three time-points up to 30 days post-stroke, reporting that microglial activation, as measured by [11C]-PK11195 binding, was first apparent at 72 h post-stroke, rising significantly within a week [94]. However, it should be noted that TSPO expression is also upregulated in reactive astrocytes, endothelial cells and infiltrating immune cells, and as such, this current method of investigating microglial activation lacks the specificity to interpret what specific cell type is contributing to the inflammation [98]. Notably, there is a clear time difference between rodent (hours) and human stroke patients (days) microglial activation, however, this is most likely due to cellular differences in microglia [99], and highlights the need for rigorous investigation to improve translatability of pre-clinical data. Corresponding with pre-clinical data, one group also discovered that microglial activation is first seen within the penumbra before later invading the infarct core (~1 week post-stroke) [92]. Taken together, these studies suggest that microglial activation occurs quite rapidly after ischaemic injury, with microglia within the infarct zone remaining activated for weeks, and perhaps even months, post-stroke.

Interestingly, another ischaemic patient study demonstrated that, although microglial activation was initially increased within the infarct zone by 6 months post-stroke, no differences in microglial activation were observed compared to controls [96]. Similar temporal dynamics were seen in an experimental study that investigated microglial and astrocytic activation after photothrombotic stroke in the rat brain; whilst microglia had extensive proliferation into the ischaemic core in the acute stage (7 days), at 60 days post-stroke, microglial activation had declined, with only a few ameboid cells visible within the core [100]. This is consistent with a recent study that documented peak microglial activation in the peri-infarct region at 7 days following photothrombotic stroke in mice, before shifting to a less activated state by day 84 [101]. These studies suggest that, although there appears to be prolonged microglial activation in the infarct core within the first 1-2 months of stroke, beyond this point microglial activation appears to return to normal. In contrast, a non-human primate study investigating the function of microglia/macrophages after ischaemia demonstrated an increased presence of cluster of differentiation 68 (CD68)-expressing microglia/macrophages in the infarct lesion in both the subacute (1–4 weeks) and chronic (4 and 20 months) stages post-stroke, compared to the acute stage (3 and 24 h) [102]. Surprisingly, CD68-expressing cells were colocalised with transforming growth factor beta, a cytokine with neuroprotective functions, suggesting that CD68-expressing microglia/macrophages may be exerting anti-inflammatory effects. Therefore, it is possible that, in the chronic stage of ischaemic stroke, activated microglia that remain in the infarct zone are working towards tissue repair [102].

While the majority of experimental studies to date give clear evidence of the positive role of anti-inflammatories as stroke treatments, thereby demonstrating that microglia have a key role in the exacerbation of tissue damage post-stroke, microglia do not only have cytotoxic functions after ischaemic injury. In line with their immunosurveillance role under normal conditions, they can phagocytose invading micro-organisms and debris, as well as promote tissue repair by secreting wound-healing factors [89]. Particularly, Lalancette-Hebert et al. and Szalay et al. have both independently demonstrated that conditional removal of microglia (using genetic and pharmacological approaches) exaggerates the severity of tissue injury around the infarct, thus highlighting the importance of microglia in brain repair after stroke [103,104]. In line with this, transplantation of exogenous microglia was found to improve functional and behavioural recovery after MCAo in rats [105,106]. In support of this, Narantuya et al. found that after human microglial cells were transplanted into rats 48 h after ischaemia, rats exhibited significant functional recovery and a significant reduction in both infarct volume and the number of apoptotic cells within the infarct core and penumbra at 7 and 14 days post-injury [106]. In addition, transplanted microglia up-regulated expression of neurotrophic factors, such as glial cell-line derived neurotrophic factor and brain derived neurotrophic factor, as well as the anti-inflammatory cytokines IL-4 and IL-5 [106]. Taken together, it is clear that the role of microglia in the post-stroke neural environment is complex, having both beneficial and detrimental effects in the different post-stroke phases. These dual properties are further complicated by the multiple activation phenotypes of microglia. As such, it is likely that complete inhibition of microglia may impede recovery after stroke; however, specific targeting may be more beneficial.

Although inadequate for the accurate description of their complex functions [107], microglial cells can be activated and loosely classified into two phenotypes: classically activated (M1) and alternatively activated (M2). Broadly speaking, the M1 phenotype of microglia is typically considered to be destructive, as they release pro-inflammatory cytokines such as TNF-α, IL-1β, IL-12 and IL-6, as well as producing ROS and NO, all of which exacerbate brain tissue damage [62,108,109,110]. Conversely, the M2 phenotype of microglia is typically considered to be anti-inflammatory, releasing anti-inflammatory cytokines which prevent inflammation and promote tissue repair [108,109,110]. In a study on microglial polarisation after MCAo in mice, M2-type microglia were observed in the peri-infarct region as early as 1 day following ischaemia, peaking between 3–5 days, and decreasing from day 7. Contrastingly, the M1-type microglia gradually increased from day 3 and remained elevated up to 14 days after ischaemia [111]. Similar findings were also observed in mice in a permanent MCAo study, suggesting that early on (≈ hours-days) after ischaemia, the primary microglial cell type is the neuroprotective M2, whereas, in the later stages (>7 days post-stroke), there is a dramatic shift towards the cytotoxic M1 phenotype [112]. Indeed, using immunofluorescent staining, Suenaga et al. revealed that in both young (2 month) and aged (18 month) mice, M2 expression peaked around 7 days post-stroke, whereas M1 expression peaked at 14 days [113]. As expected, given the negative effect of age on neuroinflammation following stroke [114,115], aged mice exhibited reduced numbers of M2-type microglia compared to young mice. Notably, M2 expression also exhibited a strong positive correlation with favourable neurological outcomes following stroke [113].

This suggests that the reason why aged individuals suffer worsened outcomes post-stroke [116,117] may be due to differences in microglial polarization, such that reducing the number of M1 microglia and promoting the transition to M2 after stroke may achieve better therapeutic effects than simply targeting all microglial cells. Consequently, various experimental studies have demonstrated a reduction in infarct volume [118,119,120,121,122,123,124,125], an increase in angiogenesis and neurogenesis [118,119], as well as decreased cognitive impairment [124] when M2-type microglia were enhanced after stroke. Specifically, a study investigating the neuroprotective potential of Fingolimod, an immunosuppressant, after cerebral hypoperfusion in mice found that treatment reduced cognitive dysfunction and white matter injury by shifting microglia polarization toward the M2 phenotype. Such findings suggest that the M1 microglia phenotype may play a role in inducing cognitive decline post-stroke [126]. As such, elucidating the mechanisms of microglial polarization long-term after ischaemia, particularly in the clinical setting, may provide insight into therapeutic strategies for targeting the correct microglial phenotype, at the relevant time post-stroke, in order to improve stroke outcomes.

### 3.2. Astrocytes

Astrocytes are the main glial cell of the brain and work to regulate ions and neurotransmitters, modulate the BBB and protect the neurovascular unit of the brain [85,127]. Just like microglia, astrocytes become activated within hours following cerebral ischaemia [128,129,130] and can exert both deleterious and beneficial functions within the brain [127]. Referred to as reactive astrogliosis, after injury, activated astrocytes show hypertrophy and proliferation, as well as an increased expression of intermediate filament proteins such as glial fibrillary acidic protein (GFAP), vimentin and nestin [127,130,131]. Indeed, astrocytes in the penumbra proliferate to the ischaemic border and secrete extracellular matrix molecules (ECM) to form a glial scar [85]. Within the glial scar, reactive astrocytes express inhibitory molecules, such as chondroitin sulphate proteoglycans (CSPGs), which various pre-clinical studies have shown inhibit axonal extension and regeneration after CNS injury [132,133,134]. In particular, Huang et al., found that GFAP positive astrocytes co-expressed CSPG and formed a thin border surrounding the ischaemic core, suggesting the presence of a CSPG-containing glial scar in the human brain after an ischaemic injury, limiting axonal regeneration and recovery post-stroke [135]. On the other hand, the glial scar functions to effectively “wall off” the necrotic infarct core from the surrounding viable tissue to limit the spread of inflammation and try and prevent further tissue damage [136,137,138]. As such, although the glial scar can be neurotoxic, it can also be beneficial and may enhance neuroprotection after ischaemic injury. Indeed, a number of studies are now investigating modulating the glial scar as a mechanism to enhance recovery following stroke [139,140,141,142].

Like microglia, astrocytes also exhibit two functional phenotypes with respect to pro-or anti-inflammatory gene expression and their concomitant neurotoxic and neuroprotective capabilities [143,144]. Classically activated (A1) astrocytes exert neurotoxic effects by releasing pro-inflammatory molecules [145,146], while alternatively activated (A2) astrocytes provide neuroprotection by secreting anti-inflammatory mediators [146,147]. Concordant with earlier studies [145,148], Jang et al. demonstrated that both lipopolysaccharide (LPS) and interferon-γ (IFN-γ) stimulated astrocytes to the classically activated phenotype, so that they showed increased NO production and TNF-a expression, as well as being toxic to neurons [143]. In contrast, IL-4 and IL-10 were discovered to be key stimuli for alternatively activated astrocytes and, in fact, mediated neuroprotective effects against excitotoxic or oxidative insults [143]. Interestingly, lipocalin-2 (LCN2), an inflammatory protein, has been shown, both in culture and in a mouse neuroinflammation model, to regulate astrocytic phenotypes and their intracellular signalling pathways by promoting classical pro-inflammatory activation and inhibiting a signalling pathway involved in alternative astrocytic activation [143]. This suggests that LCN2 is a key modulator of astrocytic polarization and could be a key target for therapeutic intervention. In line with this, a study investigating the effects of astrocytic polarization in LCN2 knockout mice discovered that lack of LCN2 modulation resulted in no classically activated astrocytes, a reduction in infarct volume and improved neurological functions following MCAo [149]. As astrocytic polarization is a relatively new concept, especially in terms of its role in acute neuroinflammation post-stroke [149], it is possible that, with future investigation, a novel therapeutic target may be revealed [144].

### 3.3. Peripheral Immune Cells

Both microglial and astrocytic activation can increase BBB permeability through upregulation of adhesion molecules and MMPs, which subsequently allow adherence, accumulation and infiltration of peripheral immune cells into the infarct zone, and degradation of the ECM components, respectively [71,150]. Following stroke onset, leukocytes infiltrate into the CNS, where they release pro-inflammatory cytokines, destructive proteases and ROS at the site of injury, resulting in further degradation of the brain tissue and subsequent increase in the infarct area [70,72,151]. Neutrophils are the earliest (within hours) leukocyte subtype to infiltrate into the ischaemic lesion, and may exacerbate neuronal injury by secreting cytotoxic substances, pro-inflammatory mediators, MMPs and adhesion molecules [152]. In support of this, clinical studies have confirmed that neutrophils infiltrate and accumulate in significant numbers within the infarct core after an ischaemic stroke, and that this accumulation correlates both with the extent of the neuronal damage and poor neurological outcome [153,154,155]. Moreover, several experimental studies have demonstrated reduced infarct volume and improved neurological outcome with inhibition of neutrophil infiltration [156,157,158]. However, all clinical trials targeting neutrophil recruitment to date have been unsuccessful due to lack of neuroprotective efficacy and unfavourable side-effects [80,159,160], suggesting that a greater understanding of the contribution of neutrophils to the ischaemic injury cascade is still needed.

Experimental stroke studies have shown that lymphocytes infiltrate into the brain at later stages (2–3 days) than neutrophils [161,162]. Similar to neutrophils, lymphocytes are also sources of pro-inflammatory mediators and cytotoxic substances [151,152], although they are considered to contribute more chronically to ischaemic tissue damage then neutrophils. Clinical studies show that lymphocytes have strong pro-inflammatory and damaging properties, with a study by Nadareishvili et al. demonstrating that levels of circulating lymphocytes correlate to an increased risk of recurrent stroke and death [163]. Contrastingly, a study that recreated ischaemic-like injury in cultured primary neurons highlighted neutrophil-specific neuronal death, with lymphocytes not shown to be neurotoxic [164]. As such, whether lymphocytes are harmful or protective in the acute post-stroke environment is still unclear. Whilst the specific role of lymphocytes after ischaemia is still a matter of debate, blocking lymphocytes from infiltrating into the ischaemic brain decreased the severity of the lesion, suggesting that, like neutrophils, lymphocytes are largely detrimental following ischaemic lesions [165]. Specifically, T lymphocytes are considered to be the key lymphocyte subtype driving inflammation after ischaemic stroke, with B lymphocytes thought to play a lesser role [166]. As such, several experimental studies evaluating T lymphocyte deficient mice consistently reported a decrease in infarct volume and improved functional outcomes compared to that of wild type mice after transient focal ischaemia [166,167,168].

## 4. Chronic Neuroinflammation in Stroke

Despite the growing body of literature on the effects of neuroinflammation in the acute post-stroke environment, to date investigation of the inflammatory reaction in the chronic phase post-stroke remain scarce. Pascotini et al. found that serum levels of TNF-α were increased in patients six months following stroke, positively correlating with pro-apoptotic markers caspase-8 and caspase-3, suggesting that neuroinflammation may drive continued cell death following stroke [169]. Similarly, higher levels of both TNF-α and IL-1β in the serum of stroke patients were found to correlated with poor stroke outcomes one year after stroke [170]. However, despite this prolonged presence of inflammatory cytokines within the serum, various studies have demonstrated that both microglial and astrocytic activation within the infract core declines over time [96,100], with no microglial differences seen between stroke patients and controls at 6 months following stroke [96]. It may be that, at chronic time-points post-injury, the neuroinflammatory response has spread beyond the initial site of the infarct to more distal brain areas, such as the thalamus, hippocampus and basal ganglia. In support of this, a PET and MRI study demonstrated microglial activation in distal areas of the ipsilateral hemisphere, such as the thalamus, at later time points (28 days and 150 days) following stroke, compared to the early increase seen in the infarct zone (3 days) [92]. Moreover, Price et al. discovered that whilst microglial activation in the infarct zone decreased at 3–4 weeks, an increase was noted in ipsilateral distal regions, as well as in the contralateral hemisphere [94]. Similar results have been noted in other neuroimaging patient studies [96,171], as well as various pre-clinical stroke studies [172,173]. Particularly, a rat study investigating microglial activation after permanent MCAo demonstrated that, although inflammation had ceased in the primary infarct site, an increased inflammatory response was observed in the ipsilateral thalamus at 7 months following stroke [174]. It is likely that this secondary peak in neuroinflammation is exacerbated by the concept of ‘inflammaging’. The term inflammaging alludes to the long-term condition of low-grade stimulation of the immune system, which essentially primes organisms to be more susceptible to inflammatory changes, such as those seen following stroke [175,176]. Suggesting that this secondary peak in neuroinflammation that is seen months after the initial insult may be exacerbated by a heightened inflammatory environment seen with ageing. As such, it is likely that chronic neuroinflammation is a key factor in the development of various chronic neurological complications post-stroke as well as in the ongoing degeneration of neuronal tissue.

## 5. Neuroinflammation in SND

**Figure 1:**
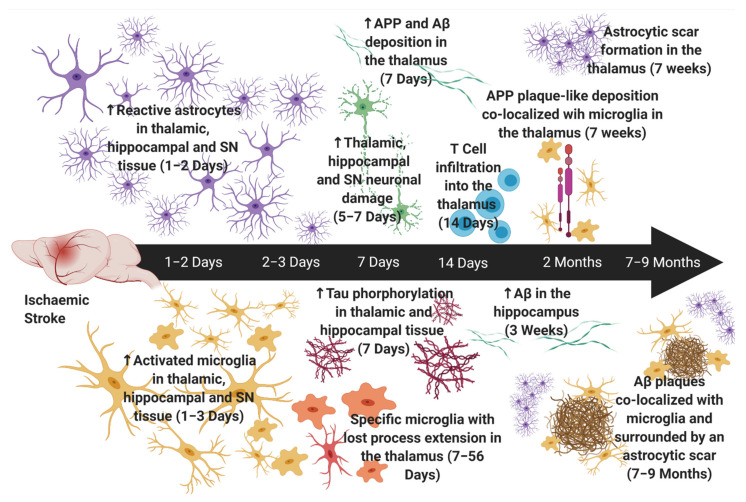
Neuroinflammation in Secondary Neurodegeneration following Experimental Stroke. Astrocytes are first increased within the thalamus, hippocampus and substantia nigra (SN) at ~one to two days post-stroke [32,185,186]. Astrocytic scar formation is first apparent in thalamic nuclei at seven weeks post-stroke [51]. Activated microglia are first increased within the thalamus, hippocampus and SN at one to three days post-stroke [177], with specific microglia (loss of process extension with intact phagocytotic functioning) seen in the thalamus at seven days and up to fifty-six days post-stroke [173,202]. Neuronal damage is noted after glial reactivity (~five to seven days) [185,186,199]. Tau phosphorylation in the thalamus [46] and hippocampus [48], and Aβ and APP deposition in the thalamus, is observed at seven days post-stroke [43]. Conversely, Aβ in the hippocampus is not apparent until three weeks post-stroke [47]. T cell infiltration into the thalamus is observed at fourteen days post-stroke [184]. APP deposition adopted plaque-like morphology and was colocalized with microglia at seven weeks post-stroke and Aβ plaques were colocalized with microglia at seven months [174] and was surrounded by an astrocytic scar at nine months post-stroke [43]. ‘↑’ denotes an increase. Created with BioRender© (https://biorender.com) (accessed on 30 November 2021).

Neuroinflammation has been implicated in SND, as demonstrated by an increase in resident inflammatory cells, such as microglia [174,177,178,179] and astrocytes [180,181,182,183], as well as infiltrating peripheral inflammatory cells, such as T cells [184] (Table 1). Most interestingly, various studies have reported a spike in inflammation preceding neuronal damage in the thalamus, hippocampus and the substantia nigra (SN), a critical part of the basal ganglia (Figure 1) [51,177,182,185]. Specifically, an earlier study by Ross & Ebner demonstrated reactive astrocytes in regions of the thalamic ventrobasal complex and the medial division of the posterior thalamic nuclei, with increased expression of GFAP, an intermediate filament protein, as early as 2 days after cortical injury, which preceded neuronal damage by at least 2 days following cortical ablation and 7–10 days following intracortical kainic acid injection in adult mice [182]. Similar results were also seen following transient MCAo in rats, with the presence of both astrocytic and microglial activation in the ventroposterior and reticular thalamic nuclei noted several days before the onset of neuronal loss in these regions [185]. Notably, another study investigating changes within the SN observed an increase in expression of GFAP, as well as CD45-positive microglia, as early as 24 h following MCAo in mice, whereas neuronal death was not seen in the region until 6 days post-stroke [186].

These results suggest that neuroinflammation is not only associated with neuronal damage in areas of SND but that is also likely to be a key driver in the neurodegenerative process. In line with this, a rodent study 7 months following permanent MCAo demonstrated a high density of activated microglia and macrophages in the thalamus that was associated with neuronal loss and colocalized with amyloid deposition [174]. Interestingly, multiple rodent studies have noted an accumulation of Aβ and phosphorylated tau associated with increased inflammatory cells as early as 1–2 weeks post-stroke within both the thalamus and hippocampus [101,187,188]. Notably, a time course study demonstrated reactive microglia in the thalamus showed an increase in iron content, suggestive of neurodegeneration, that was first localized intracellularly at 3 weeks post-stroke, but by 6 months was localized around thalamic structures, similar to Aβ plaques. Interestingly, at 7 weeks post-stroke, amyloid precursor protein (APP) was over-expressed, mainly by microglial cells, and adopted a plaque-like morphology, with iron accumulation around the plaques, indicative of neurodegeneration [51]. This suggests that microglia specifically play a key role in the accumulation of Aβ plaques and hence, distal degeneration following stroke. Moreover, one such study demonstrated that the inhibition of NMDA receptor subunit NR2B attenuated the accumulation of hyperphosphorylated tau in the hippocampus up to 14 days following MCAo in rats. Notably, this decrease in hyperphosphorylated tau was associated with improved spatial cognition [189]. Although this study did not directly investigate the associated inflammatory changes, a more recent study found that following treatment with memantine, a NMDA receptor antagonist, micro- and astrogliosis was significantly reduced at post-stroke day 14 within the thalamus [190]. Moreover, another study demonstrated that low oxygen post-conditioning provides neuroprotective effects at sites of SND, specifically the ipsilateral thalamus, after photothrombotic stroke in mice by reducing the coupling of NMDA receptors with neuronal nitric oxide synthetise, which can result in the production of NO and lead to cellular death [40]. Similar results were also seen when investigating other ion channels. Nakajima et al., demonstrated that Perampanel, a non-competitive AMPA receptor antagonist, significantly inhibited microglial activation and pro-inflammatory cytokine expression, and that this inhibition was associated with improvements in spatial cognition [191]. As such, it seems reasonable to assume that the interplay of ion channels and inflammatory cells may in some part exacerbate the neurodegeneration seen in these distal SND sites and the associated cognitive dysfunction.

### 5.1. Microglia

To date, the vast majority of the studies conducted have found evidence of microglial activation at sites of SND in both rodents and stroke patients. Specifically, microglial activation, as measured by the binding of [11C]-PK11195, was increased in the thalamus of ischaemic stroke patients between 2 months and 24 months following stroke [171]. One month following cortical ischaemia, the number of microglial cells was significantly elevated in the thalamus (lateral posterior nuclei, posterior complex and ventral posterior nuclei), with a corresponding decrease in the number of thalamic neurons in response to photothrombotic occlusion in mice [200]. In a transient MCAo rat study, activated microglia were elevated in the thalamus and striatum at day 7, peaked in the thalamus at 14–28 days, and remained elevated in the thalamus for up to 112 days post-stroke, whereas neuronal loss in the thalamus was not observed until day 14 following ischaemic injury [203]. Interestingly, the same study investigated the behavioural and pathophysiological effects of twice-a-day post-stroke treatment with (+)-naloxone, an opiate antagonist known to target microglial activation. They discovered that a week of (+)-naloxone improves short-term behavioural recovery, as measured by Bederson’s neurological assessment scale, and reduces neuronal damage in the lesioned cortex and the ipsilateral thalamus [203]. Notably, the behavioural effect of (+)-naloxone correlated with attenuation of microglial activation in both the striatum and thalamus and was not observed until day 10 post-stroke, suggesting that (+)-naloxone specifically targets SND-associated inflammation [203]. Microglial activation has also been noted in various other distal regions, such as the hippocampus and the SN. In the ipsilateral SN microglial activation was noted as early as 24 h following MCAo in mice [186]. Similarly, an earlier study in rats demonstrated increased microglial activation in both the ventroposterolateral and ventroposteromedial thalamic nuclei, as well as the hippocampus, by day 2 following permanent MCAo [177]. Persistent microglial activation was also noted in the ipsilateral hippocampus up to 3 months following a photothrombotic occlusion in mice [101] Together these studies highlight the fact that microglial activation is a key feature of SND, suggesting that targeting microglial activation may attenuate neuronal damage in these areas.

Osteopontin (OPN), a cytokine-like glycoprotein that functions as an immune modulator, may work to shift microglia to a more neutral phenotype [208]. As such, Ladwig et al. demonstrated that, following photothrombotic stroke in rats, OPN treatment shifted the M1/M2 balance towards an M2 phenotype by reducing the number of M1 microglial cells within the infarct core [209]. However, in the SND related study performed by the same group, they found that typical markers of microglial polarization states were absent in sites of SND [205]. They suggested that this might be due to the milder and more chronic nature of neuroinflammation in remote areas [205]; as they only looked 8 days following stroke, it is possible that microglial polarization is more delayed in SND than that seen in the infarct core. Moreover, as recent evidence has suggested that microglial polarization is a dynamic process that is dependent on tissue- and context-specific stimuli [210], it is possible that current markers of microglial polarization are unable to detect polarization at this early timepoint in SND. As such, further research into microglial polarization in SND sites, particularly long-term, is essential in understanding the specific nature of microglia in the development of SND.

Interestingly, Kluge et al. identified a non-responsive microglial phenotype, with intact phagocytotic functioning, that was specific to the thalamus and persisted up to 2 months following stroke [173,202]. Specifically, the group demonstrated that, upon laser damage, microglia/macrophages in the thalamus lose their process extension ability, but show an increase in phagocytic functioning 14 days after photothrombotic stroke [202]. They further showed that these microglial features are specific to the thalamus, as the microglia in the peri-infarct region retained their ability of directed process extension in response to laser damage [202]. A follow-up study by the group demonstrated that this non-responsiveness starts in the thalamus at 7 days post-stroke and persists for up to 56 days and is also correlated with thalamic neuronal damage [173]. This loss of process extension has similarly been described in neurodegenerative diseases, including both Alzheimer’s and Parkinson’s disease [211,212]. Specifically, an Alzheimer’s study in APPPS1 mice (a transgenic mouse model of cerebral amyloidosis) found that microglial directed process extension and phagocytic activity were strongly impaired in mice with Alzheimer’s disease-like pathology [213]. Notably, the impairment of microglial function correlated with Aβ plaque deposition, both temporally and spatially, suggesting that with further studies into the later stages of SND a relationship between non-responsive microglia and Aβ plaques may be observed [213] This is particularly detrimental given the fact that Aβ deposition plays a key role in the development of dementia [214]. In support of this, a recent study identified a unique subtype of CD11c-positive microglia in the thalamus 28 days following MCAo in mice [207]. Using flow cytometry, the group was able to indicate molecular signatures in the post-stroke microglia that are similar to neurodegenerative disease-associated microglia [207].

### 5.2. Astrocytes

Astrocytes are activated within the hippocampus, thalamus and SN following stroke in pre-clinical models [181,185,192,193,194,195,196,197,215]. Following photothrombotic occlusion in mice, significantly elevated GFAP expression was noted within both the ipsilateral and contralateral thalami and hippocampi (CA1, CA2 and CA3 regions) at 28 days, suggesting strong astrogliosis within SND sites [181]. Similarly, 1 day after rat transient MCAo, activated astrocytes displaying thickened cell bodies and processes were strongly enhanced in the ipsilateral ventral posterior nucleus and reticular thalamic nucleus of the thalamus, a response that persisted up to 14 days post-stroke [185]. Notably, a study investigating SND in rats following MCAo demonstrated that at the earliest timepoint examined of 3 weeks post-stroke, the astrocytic reaction was observed to be widespread in the thalamus, but at 7 and 24 weeks after injury, the reaction was localized to specific thalamic nuclei (ventral posterolateral nucleus (VPL) and ventral posteromedial nucleus (VPM)), forming an astrocytic scar [51]. Together these studies highlight the fact that astrocytic activation is associated with neurodegeneration at these distal sites, suggesting that targeting this activation may attenuate neuronal damage in these areas. Interestingly, a recent Parkinson’s study has shown a new potential approach to treating neurodegeneration by converting astrocytes to new neurons that innervate into and repopulate neural circuitry in sites of degeneration [216]. Thus, further investigation into these inflammatory cells following an ischaemic stroke may uncover viable treatment targets for the ongoing degeneration of tissue.

Astrocytes have also been implicated in the accumulation of neurotoxic proteins at sites of SND [187]. Specifically, increased levels of Aβ in the thalamus were associated with a reduction in the polarisation of aquaporin 4 (AQP4) to perivascular astrocytic end-feet [187]. It is well documented that astrocytic end-feet hold the channel protein AQP4, which allows the bidirectional movement of water with the cerebrovasculature and forms a gradient for the removal of interstitial solutes, such as neurotoxic proteins [217]. Therefore, these results suggest that loss of perivascular AQP4 polarization might impair the clearance of neurotoxic proteins in the brain [187], although it is important to note that increased deposition of Aβ in the hippocampus was also seen in the absence of any changes in AQP4 [187]. This reduced clearance of neurotoxic proteins associated with impairments in AQP4 is further supported by studies investigating the glymphatic system (GS). Specifically, AQP4 is the key molecular component of the GS, as it mediates the influx of cerebrospinal fluid (CSF) into the brain tissue and efflux of interstitial fluid into the subarachnoid space [218]. As such, one such study demonstrated that the decrease in the polarisation of AQP4 to perivascular astrocytic end-feet in both the ipsilateral thalamus and SN was associated with impaired GS function 7 days after MCAo in rats [219]. The importance of this astrocytic dysfunction was further highlighted in a PSD rat model in which bilateral common carotid artery occlusion (BCCAo) was superimposed on MCAo. They demonstrated that the tau hyperphosphorylation and cognitive impairment seen in the PSD model was likely due to the lack of tau clearance from the impaired GS [220]. Although they did not directly investigate the associated changes in AQP4, in a previous study, using the same PSD model the group found delocalization of AQP4 channels that was associated with Aβ aggregation and increased astrogliosis within the thalamus and hippocampus 2 weeks following surgery [221]. Interestingly, a recent study investigating the effects of inflammation associated cell death within the ischaemic core of rats following MCAo with reperfusion, discovered that neuroinflammation following a stroke promoted the loss of BBB integrity and AQP4 polarization, Aβ accumulation, and the formation of Aβ 1–42 oligomers [222]. These studies highlight that impairment in AQP4 and the associated dysfunction of the GS play a key role in the aggregation of neurotoxic proteins in both the ischaemic core and sites of SND. As such, AQP4 could represent a potential therapeutic target to improve neurotoxic protein clearance. Specifically, another recent study targeting inflammation related astrocytic dysfunction with Tongxinluo discovered that with restoration of AQP4 polarization the associated Aβ accumulation in the ischaemic core and hippocampus was ablated [223]. Moreover, these results are supported by other recent studies which have found that targeting AQP4 is beneficial in reducing cerebral oedema [224,225]. As such, it is clear that AQP4 may be a viable target for improving the associated degeneration of these distal sites.

Interestingly, in a cortical excitotoxic lesion model, astrogliosis and increased GFAP expression were short lived in the thalamus and were undetectable by 14 days post-lesion in comparison to the primary injury site, where the astrocytic changes persisted out to 30 days [193]. However, this is in contrast to a report of a prolonged astrocytic response in the thalamus following stroke [51,192]. As such, it is possible that this short lived astrocytic response is due to the fact that neonatal rats (9 days old) were used in the study, compared to other studies which have utilised young adult rats (~6–8 weeks) [51,192], as cortical lesions are known to be more extensive in older brains [226]. Specifically, a recent rodent study investigating age-dependent disturbances and SND demonstrated that age exacerbates the severity of SND after stroke [204], suggesting that age is a critical factor in the severity of SND. Again, demonstrating a difference in the glial response with age, a study that investigated the long term effects of a photothrombotic occlusion on 10 week old mice demonstrated a peak in reactive astrogliosis in the ipsilateral hippocampus at 7 days post-stroke, which returned to basal levels by day 84 in the CA1 sub-region [101]. As such, in order to understand the underlying signals triggering the glial response and why age/infarct severity is a factor in the glial response of these distal sites, it is imperative to improve our current understanding of the mechanisms of glial activation in SND sites, particularly long-term post-stroke.

### 5.3. Peripheral Immune Cells

Until recently, only one study had reported on the involvement of peripheral infiltrating immune cells in distal regions of the brain post-stroke. In the photothrombotic stroke model in mice, flow cytometry analysis revealed that CD4^+^ and CD8^+^ T cells were significantly increased in the ipsilateral thalamus at 14 days post-stroke; however, no changes were detected in B cells, neutrophils or monocytes [184]. Positive immunostaining of CD3^+^ (T cell co-receptor) in the ipsilateral thalamus also supported this finding. Interestingly, CD3^+^ cells were clustered to areas of strong CXCR1-GFP labelling, indicating macrophage activation [184]. However, as the authors only examined a single time point of 14 days post-stroke, which is relatively early in the SND timeline, further research is needed to confirm the role of peripheral immune cells in long-term stroke recovery. It is also important to note that common microglial markers, such as ionized calcium binding adaptor molecule 1 (Iba-1) and CD11b, are not specific to microglia and could be picking up infiltrating immune cells following stroke [227]. In contrast to this, a more recent study has demonstrated migration of B cells into remote brain areas (e.g., dentate gyrus, hypothalamus, olfactory areas, cerebellum) up to 8 weeks following MCAo in mice that were associated with neurogenesis and functional recovery [228]. Interestingly, the migration of B cells into these brain areas showed a specific positive role for motor recovery [228]. This is in line with another recent study which also showed improvements in motor recovery despite the ongoing degeneration in the thalamus and hippocampus, and the associated cognitive dysfunction [101]. As such, it is clear that similar to acute neuroinflammation following a stroke, chronic neuroinflammation in sites of SND also reflects dual properties in that it can be both detrimental and beneficial.

As BBB dysfunction and the associated increase in BBB permeability drive the infiltration of peripheral immune cells, it comes as no surprise that studies are now investigating the presence on BBB permeability prior to secondary neuronal damage [41,229]. To determine the reason behind this increased BBB permeability, Li et al. sought to explore the integrity of the tight junctions in these areas following stroke. Interestingly, they found that by 24 h following MCAo, there was a decrease in the number of intact tight junctions, which function as an effective barrier between endothelial cells, and an increase in albumin levels within the ipsilateral thalamus [41]. Following on from this study, the group investigated whether administration of Netrin-1, a regulator of BBB maintenance that protects the central nervous system against inflammation, for 7 days post-MCAo in rats, would protect against BBB associated secondary injury [229]. Notably, they discovered improvements in neurological function, up-regulation of tight junction proteins and decreased levels of albumin within the ipsilateral thalamus 14 days following ischaemia [229]. These studies suggest that early on following stroke, BBB breakdown is affecting areas distal from the infract site. As such, it is important for future SND studies to investigate the temporal dynamics of BBB breakdown, and specifically how it relates to neuroinflammation, in order to understand the full extent of BBB dysfunction and the associated peripheral immune cell infiltration.

## 6. Post-Stroke Disorders & Inflammation

Chronically, stroke can lead to significant structural changes within the brain, which can result in persistent sensorimotor dysfunction [7,230], cognitive impairments [12,14], and even neuropsychiatric disorders [231]. Unlike experimental studies, the investigation of neuroinflammation in the brain is significantly more difficult in the clinical setting. Whilst neuroimaging such as PET can provide an indication of the extent of post-stroke neuroinflammation, this imaging technology is quite expensive and not as readily accessible for both researchers and patients [232]. It is because of this that peripheral blood biomarkers are more commonly used as a surrogate for the investigation of neuroinflammatory changes. Interestingly, acute neuroinflammation, as measured by serum/CSF levels of pro-inflammatory cytokines in stroke patients, has been implicated in limiting functional recovery in the chronic phase following stroke [20,233,234]. The modified Rankin scale (mRS) classifies outcomes after stroke, with poor outcome defined as moderate to severe disability and requiring assistance with activities of daily living [235]. Indeed, one patient study demonstrated that high levels of TNF-α and IL-1β in the serum at time of admission correlated with poor mRS outcomes 1 year after ischaemic stroke [170]. Another clinical ischaemic stroke study demonstrated that higher IL-6 levels 1 day post-stroke were associated with poor mRS outcomes and shorter survival times 3 months post-stroke [236]. Consistent with this, high IL-6 serum levels 6 and 72 h post-stroke correlated with poor outcome 3 months post-stroke [234].

The most common long-term deficit caused by stroke is motor impairment, which typically affects the face, arm or leg on one side of the body, affecting around 80% of stroke patients [7]. Specifically, a review on post-stroke spasticity demonstrated that spasticity seemingly got worse over time and affected a larger percentage of patients in the chronic phase (>3 months) than in either the acute or sub-acute phases [8]. Notably, a Nigerian clinical study demonstrated that patients with moderate/severe motor disability at 30 days post-stroke had markedly higher levels of serum C-reactive protein (CRP), a non-specific biomarker of inflammation, when compared to those with mild disability [237]. Stroke also commonly affects patients’ mood and behaviour, with several meta-analyses revealing that one in every three stroke survivors are affected by post-stroke depression, most commonly seen within the first year [238,239], but which may still be apparent up to 10 years post-stroke [238]. Moreover, post-stroke depression is associated with increased mortality [240], severe disability [241], anxiety [241,242], functional and cognitive dysfunction, as well as language impairments [243]. Interestingly, during a one- year study-period, serum levels of IL-6, IL-10, TNF-α, and IFN-γ were significantly higher in the post-stroke depression group relative to a control group, suggesting a relationship between immune dysregulation and post-stroke depression [244]. Fatigue is also a common long-term complication of stroke, with a two-year follow-up study by Christensen et al. demonstrating that pathological fatigue was reported by 44% of patients 3 months post-injury, and remained constant throughout the two years [17]. Similarly, 34% of post-stroke patients reported fatigue at 12 months, which was associated with both depression and anxiety, as well as attentional-executive impairment [19]. Fatigue also seems to be driven, at least in part, by chronic neuroinflammation post-stroke, with Ormstad et al. reporting that acute serum levels of IL-1β were positively correlated with fatigue at 6 months after ischaemic insult [18]. This is further supported by a more recent study which showed that fatigue may be associated with systemic inflammation, based on correlations with the serum levels of IL-6 and CRP, in community-dwelling stroke survivors up to 5 months post-stroke [245].

### Post-Stroke Cognitive Impairment and Dementia

Of all the potential long-term neurological complications following stroke, one of the most significant is the elevated risk of developing long-term cognitive impairment or dementia. Studies investigating the prevalence of cognitive impairment in stroke patients have found that it can range anywhere from 30%-70%, depending on factors such as geographical location and the diagnostic criteria used in assessment [246]. Specifically, a study of young patients 11 years post-stroke found that 50% of patients still had noticeable cognitive impairments [247]. Vascular dementia (VaD) is a progressive form of dementia caused by the chronic reduction of cerebral blood flow (CBF) to primary brain areas [248] and is the second most common form of dementia following AD, accounting for ~10% of dementia cases worldwide [249]. Although the evolution of pathology and the specific mechanisms underlying VaD are still poorly understood, it is well-documented that VaD shares similar pathophysiology to sites of SND, such as brain atrophy, neuroinflammation, accumulation of neurotoxic proteins, etc [250]. Moreover, it is well accepted that the risk of developing VaD increases with previous history of stroke [248]. Indeed, having a stroke significantly increases the risk of developing dementia within the first 6–12 months following stroke and also doubles the chances of delayed dementia onset long-term, including the AD phenotype, as demonstrated in community-based longitudinal studies [248].

The development of cognitive impairment post-stroke may be driven by neuroinflammation. A key marker used to study this clinically is serum levels of CRP, with a history of stroke associated with increased concentrations of serum CRP [251,252]. Furthermore, higher serum CRP levels have been found to be associated with post-stroke cognitive impairment (PSCI) [11,53,253]. In support of this, serum CRP levels are associated with poor cognition and functional outcome in the acute phase post-stroke [254]. Similarly, increased levels of serum CRP on admission to hospital were associated with cognitive decline one-year post-stroke, independent of the vascular risk profile of the patients [11]. These results demonstrate that stroke-induced inflammation is a major cause of long-term functional decline following stroke, and that serum CRP may be used as a prognostic indicator of stroke recovery.

Similar to PSCI, various studies have shown that, among inflammatory markers, the level of serum CRP seems to be the strongest and the most reliable predictor of PSD [255,256,257,258,259]. In the Circulatory Risk in Communities Study (CIRCS), there was a positive association between high serum CRP levels and risk of PSD, but not between high CRP levels and risk of dementia development without a history of stroke [260]. In support of this, the Honolulu-Asia Aging Study (HAAS) demonstrated that elevated serum CRP levels in midlife increased the risk of developing dementia later in life [255]. This study, however, did not stratify individuals with dementia by their stroke history, but instead included stroke as a mediating variable, and noted that 21.5% of the dementia cases in their study had suffered a stroke [255]. Importantly, for VaD, the risk increased with each increasing quartile of serum CRP [255]. This is likely due to the fact that levels of serum CRP are associated with atherosclerosis [261] and have been shown to be predictive of cerebrovascular disease [262]. As such, it is likely that PSD and VaD are particularly susceptible to high serum CRP, as they are both associated with vascular abnormalities. This was further corroborated by the Conselice Study of Brain Aging (CSBA) which demonstrated that, while high CRP in the serum of patients was associated with risk of VaD, no association was seen with the risk of AD [258]. Moreover, due to the evidence provided by these studies, and similar evidence investigating PSCI, it is fair to assume that high levels of serum CRP seen acutely post-stroke are likely predictors of the latter onset of PSD.

Whilst studies investigating the inflammatory changes associated specifically with PSD in patients remain scarce, various experimental models of VaD have noted a link between neuroinflammation and neuronal damage following cerebral ischaemia. Specifically, a variety of experimental studies have observed an increase in pro-inflammatory cytokines in the hippocampus of VaD models [263,264,265]. Particularly, Zang et al. demonstrated that rats with VaD (measured as severe neurological deficit following MCAo) had significantly higher levels of the inflammatory markers nuclear factor kappa-light-chain-enhancer of activated B cells (NF-κB), TNF-α and IL-1β in the hippocampus compared to shams, and that this increase was associated with increased neuronal damage [266]. Furthermore, another VaD study showed that rats exposed to transient BCCAo, a global ischaemic model, demonstrated increased levels of TNF-α and IL-6 in the hippocampus compared to sham animals, with this this increase associated with neuronal death and impairment in spatial memory [267]. These experimental studies are further corroborated by VaD and post-stroke patient studies, which both show increases in TNF-α, IL-1β and IL-6 within the CSF/serum that are associated with cognitive decline [14,170,233,268,269]. Interestingly, one study investigating post-mortem VaD brains also found increased levels of TNF-α and IL-1β in the hippocampus which were associated with neuronal death in the region [269]. As such, all these studies suggest a key relationship between pro-inflammatory cytokine release and the neuroanatomical disruptions we see associated with PSD.

Although the mechanism behind this relationship still remains unclear, a study utilising a transgenic AD mouse model reported that peripheral TNF-α indirectly impacts the progression of amyloid pathology by regulating peripheral inflammatory cells, such that it promoted the deposition of Aβ [270]. As such, various VaD studies have demonstrated that increased levels of Aβ deposition and cerebral amyloid angiopathy [269,271,272]. Specifically, in a study of surgical biopsies exhibiting cerebral and cerebellar infarctions, amyloid deposition was significantly more common in samples showing infarction than in controls with non-vascular lesions [271]. Moreover, a study investigating a post-mortem VaD brain compared to a healthy brain demonstrated the presence of large Aβ deposits in the hippocampus of the VaD brain, in contrast to small deposits observed in the hippocampus of the healthy brain, probably related to the advanced age of the healthy subject [269]. Interestingly, the researchers also discovered elevations in TNF-α and IL-1β in the VaD brain, which were associated with neuronal death [269], suggesting a key link between pro-inflammatory cytokines and amyloid pathology within the hippocampus of VaD.

Thus, these pro-inflammatory cytokine increases may be directly influencing amyloid pathology within the hippocampus, such that reducing the neuroinflammatory response following stroke may attenuate the latter onset of dementia. In line with this, various studies have shown that manipulating inflammatory molecules or their pathways in experimental stroke models results in significant alterations in dementia pathology [273,274,275,276]. Specifically, the aforementioned study by Zang et al. found that Nimodipine, a calcium channel blocker, reduced the levels of NF-κB, TNF-α and IL-1β, improved the symptoms of cognitive impairment, increased CBFand alleviated neuronal injury in the hippocampus of rats [266]. These findings were further supported by another experimental study, which found that pre-treatment with vanillic acid prior to transient BCCAo significantly decreased the levels of IL-6, TNF-α and terminal deoxynucleotidyl transferase dUTP nick end labelling (TUNEL) positive cells, while also increasing IL-10 levels in the hippocampus and restoring spatial memory [267]. These treatment studies further highlight the clear association between hippocampal cytokine levels and their effect on the neuronal damage associated with cognitive dysfunction. As such, investigating the levels of these cytokines in the hippocampal tissue, and their role in the pathological mechanisms underlying PSD specifically, is likely important for increasing our current understanding of PSD and the evaluation of different treatment interventions.

## 7. Anti-Inflammatories for the Treatment of Ischaemic Stroke

Although the stroke field has seen significant advancements in reperfusion therapy using both pharmacological thrombolysis and mechanical thrombectomy, there is still a substantial need for therapeutic agents to reduce the level of brain damage before and after reperfusion, as well as further improve functional outcome [277,278]. Over the years, researchers have sought to combine both reperfusion and neuroprotective therapies with the view to revolutionising the treatment of stroke. Neuroprotective therapies refer to any agent(s) that modulate the neuronal and glial damage to reduce the extent of ischaemic brain injury [279]. As neuroinflammation plays a key role in the progression of ischaemic brain damage, as well as in the development of a range of post-stroke disorders, inflammatory mediators have been an important target for various neuroprotective agents (Table 2).

### 7.1. Acute Anti-Inflammatory Strategies

#### 7.1.1. Minocycline

A readily investigated anti-inflammatory treatment in stroke is Minocycline, a tetracycline family antibiotic, with pre-clinical studies showing that it down-regulates microglial activation, decreases inflammatory cytokine (IL-1β, TNF-α and IL-6) expression, inhibits T cell activation and decreases MMP (MMP-9 and MMP-2) activity [296,297]. Promisingly, in an open-label, evaluator-blinded study, ischaemic stroke patients treatment with oral Minocycline (200 mg dose for 5 days), had significantly better outcomes than controls [280], however, the following phase IV clinical trial was terminated for futility [285]. Fagan et al. have since performed an open-label, dose-escalation study on Minocycline to investigate its safety, tolerability and pharmacokinetics in acute ischaemic stroke patients [281]. In this study, Minocycline was administered intravenously within 6 h of stroke onset at doses of 3, 4.5, 6, and 10 mg/kg daily for 3 consecutive days. The study showed tolerability of Minocycline at all the studied doses up to 90 days following administration, with only one case of dose-limiting toxicity (toxicity-severity that limits the ability to treat the patient at the planned dose) seen for the 10 mg/kg dose [281].

Pre-clinical studies found that Minocycline has anti-inflammatory effects, with two papers published in 2011 and 2012, investigating the effects of Minocycline treatment on MMP-9 and IL-6 [282,283]. The non-randomized, dose-escalation clinical trials conducted by Switzer et al. demonstrated that Minocycline significantly reduces the plasma levels of MMP-9 and IL-6 24 h after ischaemic stroke, indicating that Minocycline has anti-inflammatory actions. Similarly, in 2012, Padma Sirvastara and colleagues performed a single blinded, open-label study to investigate the neuroprotective effects of Minocycline in patients with ischaemic stroke out to 90 days following administration. In this study, patients who received Minocycline treatment showed improvements in neurological deficits, as measured by an improvement in mRS and National Institute of Health Stroke Scale (NIHSS) scores [284]. More recently, an open-label, evaluator-blinded, clinical study showed similar results in that patients with acute ischaemic stroke who received oral Minocycline 200 mg/day for 5 days had better neurological function than controls as measured by improvement in NIHSS scores [287]. However, not all literature to date has been concordant. In a study conducted by Kohler et al., different results were reported [286]. This randomized, multicentre, open-label, blinded, endpoint evaluation, pilot study administered Minocycline intravenously at the dose of 100 mg every 12 h for up to five doses. The results suggested that, although Minocycline is well tolerated in patients with ischaemic stroke, it does not produce any significant improvement in stroke recovery [286]. Similarly, in a more recent clinical trial investigating the pharmacokinetic, anti-inflammatory and safety profile of Minocycline after intracerebral haemorrhage (ICH), although Minocycline treatment was found to be safe, no differences in inflammatory biomarkers, haematoma volume, or cerebral oedema were observed [88]. Nevertheless, it was noted in both studies that a small sample size and prolonged enrolment window may have affected the study outcomes. Specifically, in the study by Kohler et al. the mean time to treatment was 10.7 h [286], whereas in the ICH study the time to treatment was within 24 h of symptom onset [88]. These prolonged time windows threaten the efficacy of the trials, as they are not able to determine if Minocycline may have proved beneficial if administered more acutely. In general, Minocycline has been shown to produce neuroprotective effects when treatment is initiated within 24 h of stroke in both experimental and clinical stroke studies [298]. However, the timing of treatment initiation is an important consideration due to the multi-phasic role of neuroinflammation (beneficial and detrimental) in brain recovery and the delayed nature of SND. As such, in order to accurately determine the neuroprotective effects of Minocycline, more extensive clinical trials, targeting specific stroke-induced neuroinflammatory events, including acute inflammation and SND-associated inflammation, should be performed.

#### 7.1.2. Fingolimod

FTY720 (Fingolimod), a known sphingosine-1-phosphate (S1P) receptor agonist, recently highlighted in the stroke field as a promising treatment which exerts strong anti-inflammatory effects [299], such as preventing the release and infiltration of lymphocytes into the brain, as well as modulating microglial polarization [126,300,301]. Interestingly, FTY720 has also shown promise in clinical ischaemic stroke studies, with two studies demonstrating improved clinical outcomes and reduction of infarct expansion up to 3 months post-stroke [288,290]. Specifically, Zhu et al. performed a multi-centre, randomized trial on FTY720 in combination with a recombinant TPA using a dose of 0.5 mg daily for three consecutive days in 22 ischaemic stroke patients [290]. Patients who received combination FTY720 and rtPA treatment exhibited lower levels of circulating lymphocytes, significantly smaller infarct volumes, less haemorrhagic transformation, and better neurological outcomes, as measured by both the mRS and NIHSS [290]. Interestingly, in a single centre, open-label, evaluator-blinded, parallel-group clinical pilot trial, Fu et al. also showed the efficacy of FTY720 for stroke patients who were not eligible for tPA treatment [288]. Just as in the previous trial, they reported that patients in the FTY720 group exhibited lower levels of circulating lymphocytes and better neurological recovery, as measured by lower mRS and NIHSS scores [288]. In addition, the effect of FTY720 on ICH has also been reported in a two-arm, evaluator-blinded, case-control clinical trial, in which the same research group investigated eleven patients assigned to the FTY720 group (0.5 mg/day oral intake for 3 consecutive days) [289]. Promisingly, ICH patients treated with FTY720 exhibited a reduction in neurological impairment, as measured by mRS and NIHSS scores, that was also associated with lower circulating lymphocyte counts. Taken together, current clinical data suggests a beneficial role of FTY720 in preventing secondary tissue progression and promoting neurological recovery in acute ischaemic stroke, both with and without tPA thrombolysis, as well as following ICH. This suggests that, with further research, specifically into the mechanisms of long-term recovery, FTY720 may represent a novel treatment to improve stroke outcomes.

#### 7.1.3. Natalizumab

Natalizumab is a humanized CD49d antibody that works to reduce leukocytic infiltration into the brain by blocking the α4-integrin that is expressed on the surface of lymphocytes and monocytes and facilitates their adhesion to the endothelium [302]. The effectiveness of targeting a4-integrin as an ischaemic stroke treatment has yielded mixed outcomes in pre-clinical ischaemic stroke models. While some studies have shown that a4-integrin antibody treatment reduces peripheral immune cell infiltration, resulting in a subsequent reduction in infarct size [165,303], others have failed to show efficacy with treatment [304,305]. Despite these mixed results, in 2015 Elkins et al. undertook a multi-centre, double-blind, placebo-controlled, randomised, phase two study (ACTION) on the safety and efficacy of Natalizumab over 90 days in patients with acute ischaemic stroke [291]. A single dose of Natalizumab (300 mg) was given to 76 ischaemic stroke patients within 9 h of stroke onset. Although the single dose of Natalizumab was not effective in reducing MRI-defined infarct growth or improving neurological function in patients with acute stroke, the therapy was regarded as safe and provided global clinical gains on the Stroke Clinical Impact-16 scale and in cognitive function [291]. Based on these findings, a second clinical trial (ACTION II) was started in 2016 to evaluate the efficacy of a higher dose of Natalizumab. Disappointingly, the follow-up trial did not meet either its primary or secondary endpoints and, as a result, the company decided not to pursue further development of Natalizumab as an acute ischaemic stroke treatment [292].

#### 7.1.4. IL-1Ra

Interleukin-1 receptor antagonist (IL-1Ra) (Anakinra) is an anti-inflammatory protein used clinically to treat rheumatoid arthritis and is considered a promising therapy for stroke treatment [306]. In a systematic review investigating the efficacy of IL-1Ra in pre-clinical models of stroke, administration of IL-1Ra treatment was determined to be beneficial, with a vast majority of studies showing that rodents exhibited reduced infarct volume and/or improved neurobehavioral outcomes following treatment [306]. As such, in 2005, a randomised, double blind, placebo-controlled study of IL-1Ra in acute stroke patients was undertaken by Emsley et al. with the aims of assessing safety, efficacy and biological activity up to 90 days post-administration [293]. In this study, patients who received IL-1Ra treatment showed no adverse events, reductions in inflammatory cytokines and improvement in clinical outcomes, suggesting that IL-1Ra treatment is safe and well-tolerated in acute stroke. Based on these findings, a randomised, placebo-controlled phase II trial examined whether administration of IL-1Ra to patients with acute stroke affected innate cellular immune responses [294]. They discovered that treatment with IL-1Ra rapidly reverses suppression of innate immune responses by leukocytes, and that this is associated with a reduction in hypothalamic–pituitary–adrenal (HPA) axis activation. The authors noted that, although the mechanisms underlying these observations, including their impact on infection rates and stroke outcome, require further evaluation in larger studies, it is plausible that reversal of peripheral immune suppression by IL-Ra may reduce susceptibility to infection, which might positively influence clinical outcome after stroke [294]. More recently, SCIL-STROKE (Subcutaneous Interleukin-1 Receptor Antagonist in Ischaemic Stroke), which was a single-centre, double-blind, randomized, placebo-controlled phase II trial of subcutaneous IL-1Ra (100 mg administered twice daily for 3 days), was undertaken in ischaemic stroke patients presenting within 5 h of stroke onset [295]. The study showed significant reductions in plasma concentrations of IL-6 and CRP for the first 3 days following treatment; however, patients did not show a reduction in disability levels at 3 months compared to the placebo group. As such, the current findings suggest that further pre-clinical and clinical studies are required to determine the overall efficacy of IL-1Ra as a stroke treatment.

### 7.2. Challenges in Current/Past Neuroprotective Therapies

Importantly, a few neuroprotective trials have yielded positive results, and as such, provide some hope that a treatment for stroke is possible. However, despite these few success stories and the success of a multitude of treatments in pre-clinical models, the vast majority of clinical trials have failed to show efficacy and, as such, viable treatment options for stroke patients are still severely lacking [277,278,279]. Multiple factors may contribute to the high failure rate associated with neuroprotective agents, including the sub-optimal design of pre-clinical studies. In many of the earlier studies, neuroprotective agents were tested only in young animals, despite the burden of stroke in older individuals clinically and the fact that research has shown that age at stroke onset significantly alters stroke pathophysiology [114,116,117,307]. Specifically, a review by Sun et al. 2020 highlights that aged animals have exacerbated neuronal damage, accelerated infarct development, and worsened neurological deficits after ischaemic stroke, compared to young-adult animals [308]. Unsurprisingly, similar results were noted in human stroke patients, where studies reported that increased age correlates with a less favourable recovery and a higher mortality rate [307,309]. Another major discrepancy in pre-clinical research is that a majority of these studies only investigate males, with very little research being undertaken on female animals or the different responses to stroke between males and females. There is no doubt that similar ischaemic insults lead to different outcomes in males and females. Specifically, in terms of immune response differences, progesterone reduces levels of pro-inflammatory cytokines, suppresses microglial activation, and adds in myelin repair [310]. Moreover, evidence has suggested that, post-stroke, the innate cellular differences between males and females allow females to tolerate cellular stress and resist inflammation-induced cell death longer than their male counterparts [310]. As such, we should not generalize pre-clinical findings, which have only been tested on males, as there is obvious sex dimorphism in the neuroinflammatory responses seen following stroke. Moreover, it is well established that stroke outcomes are worsened in those patients with co-morbidities [311]. This is particularly troubling given that one such study found that, out of 35,690 stroke patients investigated, 94.2% of them had one or more additional morbidities present [312]. One of the few studies that has investigated the effect of age and comorbidities on specific stroke treatments discovered that almost all of the therapies were less successful in the aged cohort [313,314,315,316,317]. As such, incorporating age and comorbidities into pre-clinical research is essential and will enable researchers to more closely replicate the clinical condition, to successfully target relevant pathways, to improve the clinical translatability and stroke treatment. Finally, very few pre-clinical studies to date have explored the long-term benefits of neuroprotective therapies, particularly how these treatments may influence specific aspects of stroke recovery, such as cognition and behavioural outcomes. Given the evidence of SND as a long-term outcome of stroke [318], it is likely that studies that focus solely on the acute benefits are missing key pathophysiological changes associated with their treatment. Taken together, this indicates that various factors should be taken into account when designing pre-clinical studies. As such, to improve translation, the Stroke Therapy Academic Industry Roundtable (STAIR), as well as the Stroke Recovery and Rehabilitation Roundtable, now emphasize that studies should incorporate at least two animal species, starting with rodent and followed by a gyrencephalic species, include assessment of both acute and long-term timepoints, use a comprehensive battery to assess functional outcomes and include of models of stroke with both comorbidities and advanced age [319,320,321].

In addition to the limitations associated with pre-clinical study design, the abundance of failed clinical trials to date also points towards several deficiencies in the quality or scope of the trials themselves. Particularly, a major deficiency in stroke clinical trials is the time of administration of the neuroprotective therapy, with the majority of trials administering their therapy beyond the optimal time window or in a different time window than shown to be efficacious in pre-clinical studies [322]. One such suggestion to remedy this deficiency is to implement pre-hospital administration, thus allowing the therapy to be administered as fast as possible, in order to increase the level of tissue recovery. However, the pre-hospital strategy poses its own challenges in that, within such a short time frame, it is difficult to know if the patient would even qualify for the trial [322]. Particularly, identification of stroke patients, elicitation of consent to the trial as well as treatment randomisation are just some of the challenges the pre-hospital setting poses [322]. This is where therapies targeted specifically at SND could be important to improving long-term stroke outcomes. As SND develops progressively and chronically after stroke, it provides a wider therapeutic window and, thus, greater opportunities for treatment. This raises the question whether if these therapies were considered in the context of SND, not just the acute lesion, they might be more successful. Moreover, given the extended time window of SND, is it possible to target these processes later in the sub-acute to chronic stage and potentially provide “secondary” neuroprotection?

### 7.3. Secondary Neurodegeneration Treatment Strategies

To answer this, recent efforts have been made to interrogate the benefits of secondary neurodegeneration targeted treatments or “secondary” neuroprotection. As such, several therapeutic agents have shown that manipulating specific molecules involved in neuroinflammation have been beneficial for neuronal and functional recovery following experimental stroke [198,199,205,323].

Interestingly, a recent study looking at the effect of OPN found that rats treated with OPN had decreased neuroinflammation, microglial proliferation and evidence of neurodegeneration in the thalamus 8 days after photothrombotic occlusion, suggesting that OPN is able to ameliorate SND in thalamic nuclei [205]. In line with this, Schroeter et al. demonstrated that, 14 days after permanent MCAo, OPN knockout mice exhibited unaltered infarct development at the primary injury site, but greatly increased retrograde degeneration of the ipsilateral thalamus, resulting in increased microglial activation, inflammatory gene expression and neuronal death [198], suggesting that OPN plays a key role in the attenuation of SND. Notably, another study exploring NLRP10, a nucleotide-binding oligomerization domain (NOD)-like receptors thought to play a role in regulating pro-inflammatory signalling, found that NLRP10 knockout in mice attenuated the activation of both astrocytes and microglia in the hippocampus, and that this reduction correlated with a decrease of inflammatory cytokines, including IL-1β and TNF-α, 7 days after an MCAo operation [206]. Moreover, Jiao et al. demonstrated that Edaravone, a novel scavenger of free radicals, not only reduced the number of proliferating astrocytes in the hippocampus at 3, 7 and 30 days following MCAo, but also significantly alleviated delayed neuronal death within the hippocampus of rats, as well as reduced cognitive dysfunction, as measured by spatial learning and memory capabilities [199].

Interestingly, both Fingolimod and Minocycline have also been shown to be beneficial in targeting secondary neurodegeneration, further highlighting the idea of “secondary” neuroprotection [323,324]. Specifically, two-vessel occlusion (2VO) rats were administered intraperitoneal FTY720 (1 mg/kg) for 7 consecutive weeks from post-operative day 8, which reduced the number of pro-inflammatory cytokines and Iba-1 positive cells in the hippocampus and was associated with an improvement in memory performance [324]. Similarly, mice which received Minocycline at a dose of 30 mg/kg by intraperitoneal injection for 14 days following BCCAo showed a reduction in hippocampal neurodegeneration, as well as a reduction in the number of Iba-1 positive cells [323]. Importantly, the prolonged (14 days) Minocycline treatment significantly prevented the BCCAo-associated depressive and anxiety-like behaviours, suggesting that targeting SND-associated inflammation has a direct effect on neuropsychiatric outcome following stroke [323]. As such, future Minocycline studies should seek to extend the number of days patients receive treatment. Specifically, of the acute Minocycline trials that failed to show efficacy, they not only had longer time windows to treatment, meaning they were likely unable to prevent acute stroke damage, but they also had shorter treatment timelines [88,286], compared to studies that showed success [284,287], meaning they also likely missed the chance of targeting SND, and hence could be the reason they were unsuccessful.

Another emerging approach, which has yet to be investigated in the context of stroke, is to ablate and repopulate microglia using colony stimulating factor 1 receptor inhibitor (PLX). Two recent studies in the context of traumatic brain injury have reported that the depletion of chronically activated microglia, and subsequent repopulation with new healthy microglia, significantly improved neuropathological and functional outcomes [325,326]. Within this paper, it is well documented that simply inhibiting microglia post-stroke is insufficient to significantly improve post-stroke outcomes, as microglia are known too also be beneficial. As such, it would be interesting to see how this approach would affect post-stroke recovery and, specifically, sites of SND if it was to be investigated in the context of stroke.

## 8. Future Perspectives

It is key to recognize that despite the extensive knowledge gained on the pathophysiology of stroke, we are still far from understanding the full extent of neuroinflammatory changes and responses seen following stroke. Through investigations in pre-clinical stroke models and clinical stroke, it is undeniable that the neuroinflammatory response to stroke is initiated within minutes and has lasting effects, not only within the infarct core but also in distal regions. Interestingly, in these distal regions, spikes in neuroinflammatory cells are seen sometime after the initial spike in the infarct core, but prior to the presence of degenerative proteins, suggesting that stroke outcome may in part be influenced by a persistent neuroinflammatory response brought on by the initial ischaemic injury cascade. As such, it makes sense that pre-clinical models investigating the efficacy of anti-inflammatory neuroprotective therapies have shown positive results not only in reducing the level of neuroinflammation within the brain but also improving functional and behavioural deficits. Nonetheless, given the fact that most of these anti-inflammatory molecules have failed early in clinical trials, whether these effects can be translated into stroke patients is still under debate. This suggests that the mechanisms through which inflammatory processes contribute to stroke injury clinically are still not fully understood. Several issues could explain this. One is that there is a discrepancy between the neuroinflammation seen following experimental stroke and stroke in patients. To address this, anti-inflammatory therapies should be investigated in several clinically relevant experimental models before their translation to clinical trials., including models of differing age, gender, and number of comorbidities. Furthermore, so far most of the experimental studies targeting neuroinflammation have failed to cover the repercussions of long-term inflammatory responses, specifically in these SND regions distal to the infarct core. As such, acquiring a deeper knowledge of the effect of long-term neuroinflammation could help identify appropriate therapeutic interventions for enhancing recovery in clinical trials. Consequently, modulating the inflammatory changes in these distal sites following stroke may be a viable treatment strategy to prevent the delayed loss of brain tissue, as well as the accumulation of Aβ and hyperphosphorylated tau, thereby reducing the risk of post-stroke dementia. Moreover, due to the longevity of immune responses in these areas, the number of patients able to benefit from successful treatments would severely increase, making further investigation into SND-associated neuroinflammation essential to improving the translational strength of anti-inflammatory therapies.

## Figures and Tables

**Table 1:** Summary of current experimental studies of neuroinflammation in secondary neurodegeneration.

	Strain /Species	Stroke Model	Time Points Post-Stroke	Key Findings
Ross & Ebner, 1990 [182]	Female BALB/c mice	- -Ablation of the somatosensory cortex - -Intracortical injection of kainic acid	1–120 Days	- 2 days after either injury type, astrocytes in the thalamus became reactive and expressed increased levels of GFAP, preceded neuronal loss neurons by at least 2 days following cortical ablation and by 7–10 days following intracortical kainic acid injection - Astrocytes remained reactive up to 60 days after cortical injury.
Herrera & Cuello, 1992 [192]	Male WR	Terminal pia-arachnoid vessel occlusion	1, 4, 7, 15 and 30 Days	- Increase in GFAP+ astrocytes in the thalamus evident from day 4, persisted to 30 days after devascularization. - Correlation between signs of neuronal degeneration and increased GFAP
Morioka, Kalehua & Streit, 1993 [177]	Male WR	Bipolar Coagulation (MCAo)	20 Minutes 3, 6, 18 Hours 1, 2, 3, 5, 7, 14, 21, 28 Days 5, 6, 7 Weeks	- Early microglial activation in contralateral cortex and hippocampus; present and prominent in the thalamus at 2 days, subsided after max intensity reached at 7 days.
Acarin et al., 1999 [193]	Female and Male SDR	Injection of N-methyl-d-aspartate into the right sensorimotor cortex	4 h, 10 h, 1, 3, 5, 7, 14 and 30 Days	- GFAP immunostaining first evident in the thalamus at day 1, peaked at days 3 and 5, resolved by day 14 - Astrocytic response was different in infarct core with the maximum GFAP staining seen at day 7 post-stroke, persisted at 30 days post-stroke.
Dihne & Blocak, 2001 [194]	Male SDR	Transient MCAo	1, 3, 7 and 14 Days	- Microglial activation and increased IL-6 levels in the SN at 3 and 7 days - Reactive astrocytes at 7 and 14 days - Neuronal loss first apparent at 3 days
Zhao et al., 2001 [195]	Male SDR	Permanent MCAo	1, 4, 7 and 12 Days	- Ipsilateral SN neuronal loss peaked at day 4, continued up to day 12 - Increase in reactive astrocytes in ipsilateral SN on days 4 and 7
Dihne et al., 2002 [185]	Male SDR	- -Transient MCAo - -Photothrombotic	1, 3, 7, and 14 days	- Significant neuronal loss in VPN and RTN following MCAo - GFAP immunoreactivity seen in VPN and RTN at day 1 following MCAo, no significance was noted on day 3, 7 or 14. Similar time course seen in the PT model. - 3 days after MCAo activated microglial cells were observed in the VPN and RTN and remained elevated out to 7 and 14 days. Similar time course seen in the PT model - 14 days after PT stroke, neuronal loss in the ipsilateral VPN; ipsilateral RTN displayed no signs of neuronal damage
Zhao et al., 2002 [196]	Male SDR	Permanent MCAo	1, 2, 4, 7 and 12 Days	- Increase in reactive astrocytes in the ipsilateral SN, on days 4, 7 and 12
Loos, Dihne & Block, 2003 [197]	Male SDR	Transient MCAo	1, 3 and 7 Days	- Increased TNF-α levels in ipsilateral thalamus at 1 day and SN at 3 days - Activated microglia and astrocytes in the thalamus and SN after 3 days. - Co-localisation of GFAP and TNF-α observed in the thalamus. Co-localisation of NeuN and TNF-α was observed in the SN.
Schroeter et al., 2006 [198]	Wild type and OPN KO mice	Photothrombotic	3, 7 and 14 Days	- Thalamic microglial activation was first apparent at day 7 in both groups. - At day 14, thalamic microglial activation was strongly exacerbated in OPN KO mice. - Neuronal loss in the ipsilateral thalamus, showed no difference between WT and KO mice at day 7, but was greatly exacerbated in KO animals at day 14. - Increased iNOS, IL-1β and TNF-α levels in KO mice at day 1, most pronounced at day 10 - Delayed treatment of OPN KO mice with iNOS inhibitors reduced thalamic neurodegeneration
Justicia et al., 2008 [51]	Male WR	Transient MCAo	MR: 1–7, 10, 14, 20, and 24 weeks IHC: 3, 7, and 24 weeks	- Thalamic T2 hyperintensity detected at 3 weeks after stroke, disappeared around week 7 when T2*-weighted images showed a marked hypointensity in that area. - Neuronal loss was evident in VPL and VPM at 3 weeks, remained out to 24 weeks - Astrocytic and microglial reactivity was first apparent in the thalamus at 3 days - Astrocytic reaction was localized mainly around the VPL and VPM at 7- and 24-weeks post-stroke, forming an astrocytic scar. - Microglial reactivity increased at 7 weeks (compared to 3 weeks), remained elevated at 24 weeks post-stroke. - Reactive microglia in the thalamus showed intracellular iron content at 3 weeks, by 7 weeks the iron showed a parenchymal distribution. After 6 months iron was localized around thalamic structures like Aβ plaques. - APP expression was localized as small dots in the VPL and VPM at 3 weeks, still apparent at 7- and 24-weeks - High density of microglial cells around the APP deposits.
Lipsanen, Hiltunen & Jolkkonen, 2011 [180]	Male WR (Ibuprofen-treated MCAo, MCAo, Sham)	Intraluminal filament model of MCAo	29 Days	- Both astrocytic and microglial activation increased in the ipsilateral thalamus of both MCAo groups compared to shams. Ibuprofen-treated rats showed increased microglial activation compared to MCAo. - Aβ deposits in the thalamus were increased in MCAo animals compared to shams; No differences were seen in the ibuprofen-treated rats compared to MCAo. - Increase of calcium staining was seen in the thalamus in MCAo groups compared to shams. - Significant overall MCAo group effect was seen in limb-placing, beam-walking and cylinder tests when compared to shams; No differences between MCAo and ibuprofen-treated rats.
Jiao et al., 2011 [199]	Male WR	Transient MCAo	3, 7 and 30 Days	- Neuronal loss within ipsilateral hippocampus was increased at all time-points - Treatment with Edaravone decreased the hippocampal neuronal loss. - Increased levels of IL-1β, TNF-α and GFAP+ cells in the ipsilateral hippocampus at day 3 and 7 post-stroke; Treatment with Edaravone decreased these levels. - Edaravone reduced cognitive dysfunction (Water maze task).
Rodriguez-Grande et al., 2013	Male C57/BL6 mice	Transient MCAo	4 and 24 h 6 Days	- Loss of SP in ipsilateral SN at 24 h - Increased number of GFAP+ astrocytes and CD45+ microglia in area of SP loss 24 h. - Neuronal death not observed until 6 days
Walberer et al., 2014 [174]	Male WR	Intra-arterial injection of 2 TiO2 spheres (MCAo)	7 Days and 7 Months	- Ipsilateral thalamus of all animals showed a marked hypointensity in T2*-weighted MRI, indicating iron deposition at 7 months. - Iron deposition localized in and around the phagocytic cells. - Microglia/macrophage activity restricted to the thalamus 7 months post-stroke. - High density of activated microglia/macrophages in the thalamus at 7 months; associated with a loss of NeuN+ neuronal cells and amyloid deposition. - In ipsilateral thalamus, no changes in FDG transport could be observed, thereby indicating normal tissue perfusion.
Patience et al., 2015 [181]	Male C57B/6 mice	Photothrombotic	28 Days	- Significantly increased GFAP expression in the ipsilateral and contralateral hippocampus (CA1, CA2 and CA3), hypothalamus and thalamus at 28 days
Jones et al., 2015 [200]	Male C57BL/6 mice	Photothrombotic	28 Days	- Numbers of microglial-like cells, as well as markers of microglial structural reorganization (Iba-1), complement processing (CD11b), phagocytosis (CD68), and antigen presentation (MHC-II) were all elevated in the thalamus at 28 days and were associated with neuronal loss - Stroke animals that were also exposed to chronic stress exhibited significantly lower levels of microglia and a reduced expression of CD11b in the thalamus compared to the stroke group.
Weishaupt et al., 2016 [179]	Male SDR	Injection of endothelin-1 to induce stroke in the PFC	28 Days	- Post stroke, the DMN of the thalamus, the RSA of the cortex and the IC displayed degenerating cells. - No difference was observed in NeuN+ neurons in the thalamus between stroke and controls. - Area of Iba-1 positive signal in DMN was doubled post-stroke. - Significant neuronal loss and Iba-1 positive cells in RSA post-stroke. - Area of Iba-1 signal in IC significantly higher post-stroke. - No indication of myelin loss in the IC post-stroke.
Cao et al., 2017 [201]	Male C57BL/6 mice	Transient MCAo	30 Days	- Hypothermia initiated at 3.5 hours after stroke reduced primary cortical injury and secondary thalamic injury. - Hypothermia led to a smaller thalamic lesion size, decreased neuronal loss and astrogliosis in the thalamus and less thalamic fibre loss, as well as improvement in functional outcome
Kluge et al., 2017 [202]	Male HCM	Photothrombotic	14 Days	- Microglial process extension lost within the thalamus but remained intact within the lesion site. - Microglia at both sites displayed an activated morphology and elevated levels of activation markers. - Identification of a non-responsive microglia phenotype specific to areas of SND post-stroke.
Anttila et al., 2018 [203]	Male SDR	Transient MCAo	2, 7, 14, 28 and 112 Days	- Activated microglia seen in ipsilateral thalamus at day 7 post-stroke, CD68 immunoreactivity peaked at 14–28 days and persisted until day 112 - Neuronal loss in the ipsilateral thalamus at 14 days - Thalamic astrogliosis seen from day 7 onward, persisted for up to 112 days - (+)-naloxone treatment prevented delayed neuronal death and reduced the number of activated microglial cells in the ipsilateral thalamus and promoted behavioural recovery in the 14-day period.
Jones et al., 2018 [184]	Male HCM	Photothrombotic	14 Days	- PT stroke resulted in increased Iba-1+ cells and reduced NeuN+ cells in the ipsilateral thalamus at 14 days compared to the contralateral thalamus. - Significant numbers of CD4^+^ and CD8^+^ T cells detected in ipsilateral thalamus. - Myeloid cells, neutrophils, monocytes, and B cells were not increased in the thalamus post-stroke.
Kluge et al., 2018 [173]	Male HCM	Photothrombotic	3, 7, 14, 28 and 56 Days	- Microglial processes in the thalamus remained responsive to laser-damage for up to 3 days after stroke, at day 7 microglia did not respond to laser damage and this non-responsiveness persisted for up to 56 days; response specific to the thalamus - Impairment of microglial processes extension not due to complete functional paralysis, as continuous engagement in phagocytosis was observed. - Microglial disturbances and neuronal loss appeared to overlap across time and were predominantly located within the Po and VPL. - At days 14, 28 and 56, in the thalamus microglia morphology parameters including cell number, cell area, cell radius, branch length and cell solidity were significantly different to shams.
Kluge et al., 2018 [204]	Young (2–4-month-old) and aged (22–23-month-old) Male C57BL/6J mice	Photothrombotic	7 Days and 28 Days	- Aged mice performed worse in the cylinder task and grid walk task compared to young mice at 7 and 28 days - Levels of synaptic markers in the thalamus were reduced in aged mice compared to young mice at 28 days - Increase in Aβ in the thalamus in aged mice compared to young mice - No differences seen in microglia and astrocytes with age
Ladwig et al., 2018 [205]	Male WR	Photothrombotic	8 Days	- Microglial activation and neuronal loss in thalamus at 8 days, neuronal loss restricted to areas of microglial infiltration. - OPN treatment significantly decreased neurodegeneration, inflammation, and microglial proliferation within the thalamus; no effect on infarct volume. - OPN attenuated the microglial response
Sanchez-Bezanilla et al., 2019 [187]	Male C57BL/6 mice	Photothrombotic	14 Days	- Increased neuronal loss, astrogliosis, Aβ and α-Syn accumulation in the peri-infarct region, thalamus and hippocampus - A loss of AQP4 polarity in the peri-infarct region and thalamus, but there were no significant changes in the hippocampus
Li et al., 2020 [206]	Male wild type C57BL/6 and NLRP10-knockout (KO) mice	Transient MCAo	7, 14 and 28 Days	- Following NLRP10 knockout, levels of microglia and astrocytes in the ipsilateral hippocampus were decreased compared to stroke animals. Motor deficiencies were also relieved post-stroke. - Serum derived from NLRP10-KO mice produced significantly lower concentrations of IL-6, IL-1β and TNF-α. - Significant increase of inflammatory regulators, including IL-6, IL-1β, IL-18, TNF-α, and CD68 in hippocampus; significantly decreased by NLRP10 knockout.
Cao et al., 2021 [207]	Male C57BL/6 mice	Permanent MCAo	1, 3, 7, 14, 28, 56, and 84 Days	- Neuronal loss seen in the ipsilateral thalamus up to 84 days - A unique subtype of CD11c-positive microglia was noted in the thalamus at 28 days. Using flow cytometry, the microglia was shown to have a reduced expression of Tmem119 and CX3CR1, and increased expression of ApoE, Axl, LpL, CSF1, and Cst7
Kim et al., 2021 [190]	Young (11–14 weeks) and aged (18–22 months) male C57BL/6J	Permanent MCAo	3 and 14 Days 6 Weeks 2 Years	- Increase in microglia and astrocytes within the thalamus at day 3, increased at day 14 when neuronal loss was first noted - Aged mice demonstrated reduced microgliosis and astrogliosis compared with young mice - Astrogliosis in the ipsilateral thalamus at 6 weeks post-stroke was similar to that at day 14, but by 2 years had glial scar properties
Sanchez-Bezanilla et al., 2021 [101]	Male C57BL/6 mice	Photothrombotic	7, 28 and 84 Days	- Persistent impairment in cognitive function post-stroke as measured by the PAL and VDR tasks - Neuronal loss and Aβ accumulation seen in the peri-infarct region up to 84 days post-stroke - Levels of inflammatory cells in the peri-infarct region peaked at 7 days post-stroke - Persistent neuronal loss, increased inflammatory cells and Aβ accumulation in the hippocampus up to 84 days post-stroke

Wistar rats (WR), Sprague-Dawley rats (SDR), Heterozygous Cx3CR1GFP/WT mice (HCM), middle cerebral artery occlusion (MCAo), blood-brain barrier (BBB), Osteopontin (OPN), dorsomedial nucleus (DMN), retrosplenial area (RSA), internal capsule (IC), posterior complex (Po), ventral posterior nucleus (VPN), reticular thalamic nucleus (RTN), substantia nigra (SN), ventral posteromedial nucleus (VPM), substance P (SP), paired associates learning (PAL), visual discrimination reversal (VDR) and ventral posterolateral nucleus (VPL).

**Table 2:** Existing clinical data on the immunomodulatory drugs discussed in this review: Minocycline, Fingolimod, Natalizumab, and IL-1Ra.

Drug	Year/Drug Trial	Stroke Type	Drug Onset	Administration Route	Timing	Sample Size	Main Outcome	Ref.
Minocycline	2003 to 2005	AIS	Within 24 h	200 mg Orally	Daily for 5 days	151 (*n* = 74 minocycline, *n* = 77 placebo)	Lower NIHSS and mRS, higher BI in the minocycline treated compared with control from day 7 to day 30	Lampl et al. (2007) [280]
	2008 to 2009 (MINOS)	AIS	Within 6 h	3.0, 4.5, 6.0, 10.0 mg/kg I.V.	Every 12 h for 3 days	60 (*n* = 11 for 3.0 mg/kg, *n* = 4 for 4.5 mg/kg, *n* = 4 for 6.0 mg/kg, *n* = 41 for 10 mg/kg)	Minocycline is safe and well tolerated up to doses of 10 mg/kg alone and in combination with tPA	Fagan et al. (2010) [281]
	2008 to 2009 (MINOS)	AIS	Within 6 h	3.0, 4.5, 6.0, 10.0 mg/kg I.V.	Every 12 h for 3 days	60 (*n* = 11 for 3.0 mg/kg, *n* = 4 for 4.5 mg/kg, *n* = 4 for 6.0 mg/kg, *n* = 41 for 10 mg/kg)	Lower plasma MMP-9 was seen among minocycline treated subjects in combination with tPA	Switzer et al. (2011) [282]
	2008 to 2009 (MINOS)	AIS	Within 6 h	3.0, 4.5, 6.0, 10.0 mg/kg I.V.	Every 12 h for 3 days	60 (*n* = 11 for 3.0 mg/kg, *n* = 4 for 4.5 mg/kg, *n* = 4 for 6.0 mg/kg, *n* = 41 for 10 mg/kg)	MINOS patients had lower IL-6 at 24 h compared with the separately non-treated cohort	Switzer et al. (2012) [283]
	2006 to 2008	AIS	N/A	N/A	N/A	29		
	Not disclosed	AIS	Within 24 h	200 mg Orally	Daily for 5 days	50 (*n* = 23 minocycline treated, *n* = 27 placebo)	Lower NIHSS score in the minocycline-treated compared with control on day 30 and 90, lower mRS and higher BI at 90 days also	Padma Srivastava et al. (2012) [284]
	2009 to 2012	AIS	Within 48 h	200 mg Orally	Daily for 5 days	139	Terminated for futility	Singh et al. (2013) [285]
	2010 to 2012 (PIMSS)	AIS and ICH	Within 24 h	100 mg I.V.	Every 12 h for 2 days	95 (*n* = 47 minocycline, *n* = 48 placebo)	Intravenous minocycline was safe but not efficacious	Kohler et al. (2013) [286]
	2012	AIS	Within 24 h	200 mg Orally	Daily for 5 days	53 (*n* = 26 minocycline, *n* = 27 placebo)	Lower NIHSS score in the minocycline-treated compared with control on day 90	Amiri-Nikpour et al. (2015) [287]
	2013 to 2016	ICH	Within 24 h	400 mg I.V. (First Dose) 400 mg Orally (Subsequent Doses)	Daily for 4 days	16 (8 randomised to minocycline)	No differences were observed in inflammatory biomarkers, hematoma volume, or perihematomal oedema	Fouda et al. (2017) [88]
Fingolimod	2012 to 2014	AIS	Within 72 h	0.5 mg Orally	Daily for 3 days	22 (*n* = 11 fingolimod, *n* = 11 control)	Fingolimod recipients had lower circulating lymphocyte counts, milder neurological deficits, and higher BI scores, lower NIHSS and mRS scores	Fu et al. (2014) [288]
	2012 to 2014	ICH	Within 72 h	0.5 mg Orally	Daily for 3 days	23 (*n* = 11 fingolimod, *n* = 12 control)	Fingolimod improved NIHSS, GCS, mRS and BI scores, and had lower circulating lymphocytes	Fu et al. (2014) [289]
	2013 to 2015	AIS	Within 4.5 h	0.5 mg Orally	Daily for 3 days	47 (*n* = 25 tPA alone, *n* = 22 tPA and fingolimod	Fingolimod patients had lower circulating lymphocytes, smaller lesion volumes, less haemorrhage and attenuated neurodeficits with lower NIHSS scores	Zhu et al. (2015) [290]
Natalizumab	2013 to 2015 (ACTION)	AIS	Within 9 h	300 mg I.V.	Single dose	161 (*n* = 79 natalizumab, *n* = 82 placebo)	Natalizumab did not reduce infarct growth or improve NIHSS scores, however, improvements were seen in mRS and BI scores	Elkins et al. (2017) [291]
	2016 to 2017 (ACTION II)	AIS	Within 24 h	300 and 600 mg I.V.	Single dose	239 (*n* = 77 300 mg dose, *n* = 81 600 mg dose, *n* = 81 placebo)	Natalizumab did not improve patient outcomes at either dose	Elkind et al. (2020) [292]
IL-1Ra	2001 to 2003	AIS and cortical infarcts	Within 6 h	100 mg I.V. 2 mg/hg/h infusion	Single 100 mg dose followed by 72 h of infusion	34 (*n* = 17 IL-1Ra, *n* = 17 placebo)	Reduction in total white cell count, neutrophil count, NIHSS and mRS scores and plasma CRP and IL-6 in patients receiving IL-1ra compared with placebo	Emsley et al. (2005) [293]
	2001 to 2003	AIS and cortical infarcts	Within 6 h	100 mg I.V. 2 mg/hg/h infusion	Single 100 mg dose followed by 72 h of infusion	34 (*n* = 17 IL-1Ra, *n* = 17 placebo)	Treatment with IL-1Ra reverses peripheral innate immune suppression	Smith et al. (2012) [294]
	2014 to 2016 (SCIL-STROKE)	AIS	Within 5 h	100 mg SC	Twice daily for 3 days	80 (*n* = 39 IL-1Ra, *n* = 41 placebo)	Reduced plasma IL-6 and CRP, showed no improvement in mRS scores	Smith et al. (2018) [295]

Acute ischaemic stroke (AIS), intracerebral hemorrhage (ICH) National Institute of Health Stroke Scale (NIHSS), Modified Rankin Scale (mRS), Barthel Index Scale (BI), Intravenous (I.V.), Interleukin 6 (IL-6), Matrix metalloproteinase 9 (MMP-9), Glasgow Coma Scale (GCS), C-reactive Protein (CRP).

## Data Availability

Not applicable.

## Abbreviations

AD	Alzheimer’s disease
AMPA	amino-3-hydroxy-5-methyl-4-isoxazole propionic acid
APP	amyloid precursor protein
AQP4	aquaporin 4
ATP	adenosine triphosphate
Aβ	amyloid-beta
BBB	blood-brain barrier
BCCAo	bilateral common carotid artery occlusion
CBF	cerebral blood flow
CD	cluster of differentiation
CNS	central nervous system
CRP	C-reactive protein
CSF	cerebrospinal fluid
CSPGs	chondroitin sulphate proteoglycans
ECM	extracellular matrix
GFAP	Glial fibrillary acidic protein
GS	glymphatic system
Iba-1	ionized calcium binding adaptor molecule 1
ICH	intracerebral haemorrhage
IFN-γ	interferon-γ
IL	interleukin
IL-1Ra	interleukin-1 receptor antagonist
LCN2	lipocalin-2
LPS	lipopolysaccharide
MCA	middle cerebral artery
MCAo	middle cerebral artery occlusion
MMP	matrix metalloproteinases
MRI	magnetic resonance imaging
mRS	modified Rankin scale
NF-κB	nuclear factor kappa-light-chain-enhancer of activated B cells
NIHSS	National Institute of Health Stroke Scale
NMDA	N-Methyl-D-aspartate
NO	nitric oxide
OPN	osteopontin
PET	positron emission tomography
PSCI	post-stroke cognitive impairment
PSD	post-stroke dementia
ROS	reactive oxygen species
SN	substantia nigra
SND	secondary neurodegeneration
TNF-α	tumor necrosis factor-alpha
tPA	tissue plasminogen activators
TSPO	translocator protein
VaD	vascular dementia
VPL	ventral posterolateral nucleus
VPM	ventral posteromedial nucleus
